# Increased Levels of CHI3L1 and HA Are Associated With Higher Occurrence of Liver Damage in Patients With Obstructive Sleep Apnea

**DOI:** 10.3389/fmed.2022.854570

**Published:** 2022-02-25

**Authors:** Jingyao Cai, Xing Lyu, Peiying Huang, Shisheng Li, Ruohong Chen, Zhiyang Chen, Mei Sun, Ling Zeng, Fengxi Wu, Min Hu

**Affiliations:** ^1^Department of Laboratory Medicine, The Second Xiangya Hospital of Central South University, Changsha, China; ^2^Department of Otolaryngology-Head and Neck Surgery, The Second Xiangya Hospital, Central South University, Changsha, China

**Keywords:** obstructive sleep apnea-hypopnea syndrome, liver damage, Chitinase 3-like protein 1, hyaluronic acid, biomarker, liver fibrosis

## Abstract

**Background:**

Obstructive sleep apnea-hypopnea syndrome (OSA) may cause liver fibrosis, and liver fibrosis serum biomarkers plays an important role on the diagnosis of liver fibrosis. In addition, this study aimed to observe the changes of 4 serum markers and Chitinase 3-like protein 1 (CHII3L1) levels in OSA patients with different disease severity and explore their interactions. And then, we examined whether intermittent hypoxia (IH) exposure can activate hepatic stellate cell.

**Methods:**

74 OSA patients in Second Xiangya hospital from January 2021 to October 2021 was selected and categorized into mild, moderate, and severe groups according to AHI. In addition, 20 subjects were selected as the control group. Serum levels of liver fibrosis markers were determined by electrochemiluminescence immunoassay. Hepatic stellate cells were exposed to intermittent IH or normoxia (RA). Results were analyzed using the SPSS software.

**Results:**

There was a significant increase in serum hyaluronic acid (HA), collagen type IV (CIV) and CHI3L1 levels in OSA patients compared with control group. Specifically, serum liver fibrosis markers HA, CIV and CHI3L1 levels were positively correlated with apnea-hypopnea index (AHI), but negatively correlated with the lowest saturation oxygen (LSaO_2_) respectively. The LX-2 cells (human hepatic stellate cell line) exposed to IH showed significant increases in fibrotic protein expression.

**Conclusion:**

OSA might either directly or indirectly trigger or exacerbate liver fibrosis, possibly via IH-related pathways.

## Introduction

Obstructive sleep apnea-hypopnea syndrome (OSA) is a common disorder that affects all age groups, especially middle-aged and elderly people (1). It is characterized by intermittent episodes of upper airway obstruction during sleep, resulting in increased respiratory effort, recurrent decreased arterial oxygen saturation and sleep disruption (2). Various evidence suggests that the prevalence of OSA is increasing worldwide, which may be due to increased living standards and rising obesity rates (3). Therefore, it is of great interest to explore the parthenogenesis of OSA, which could lay the foundation for effective treatment of OSA.

Epidemiological studies have shown that OSA subjects have more than a 5-fold higher risk of developing liver disease compared to non-OSA subjects (4). There are some experimental and clinical data have suggested that OSA may contribute to the development and exacerbation of liver fibrosis, which occurs in response to any etiology of almost all chronic liver diseases (5, 6). Biopsy has long been touted as the gold standard for the assessment of liver fibrosis. However, it has been limited by its invasiveness, cost, associated complications, sampling variability, and potential risks of clinical application (7). In view of these limitations, it has been suggested that non-invasive methods should be developed to greatly facilitate the clinical management of liver fibrosis and reduce the need for biopsy. In recent years, serum marker levels have been introduced as a non-invasive method to assess liver fibrosis (8). Hyaluronic acid (HA), procollagen type III N-terminal peptide (PIIINP), collagen type IV (CIV), and laminin (LN) are four common markers of liver fibrosis. Their diagnostic properties have been confirmed by many studies (9). However, it is difficult to determine the stage of liver fibrosis based on the results of these four serum fibrosis markers alone.

Chitinase 3-like 1 (CHI3L1) is a novel biomarker to diagnosis of liver fibrosis. Based on RNA sequencing data, CHI3L1 expression in liver is higher than that in other tissues like heart, brain, kidney and so on (9). In other words, CHI3L1 is a highly liver-enriched gene which may be a good marker of liver disease (10). Immunohistochemical studies have shown that CHI3L1 is expressed in fibrotic areas of the liver (11, 12). And it also has a strong correlation with the degree of liver fibrosis. Huang et al. showed CHI3L1 outperforms CIV, HA, LN and PCIII in diagnosing liver fibrosis (13). Based on these supporting evidence, serum CHI3L1 has been assumed to be a useful biomarker for liver fibrosis and prognosis (14).

However, the correlation between OSA and liver fibrosis is not well defined, but chronic intermittent hypoxia (CIH), as quantified by oxygen saturation, has been shown to be an important causal factor (15, 16). Hepatic stellate cells (HSCs) are now considered to be one of the key cell types involved in the progression of liver fibrosis and associated pathophysiological and clinical complications (17). Following liver tissue damage, hematopoietic stem cells undergo an activation process characterized by a phenotype of proliferation, motility, contraction, and synthesis of ECM components to promote the formation of liver fibrosis (18).

Therefore, the aim of our study was to investigate changes in the levels of four serum markers and CHII3L1 in OSA patients with different disease severity and to explore their interactions. We then examined whether CIH exposure could activate hepatic stellate cells. These results will contribute to a better understanding of the pathogenic mechanisms of OSA.

## Materials and Methods

### Human Subjects

Ninty-four Patients Were Recruited at the Second Xiangya Hospital of Central South University (Changsha, China) between January 2021 and October 2021. The Diagnosis of OSA Was Determined According to the Relevant Clinical Practice Guidelines by American Academy of Sleep Medicine (AASM) (19). A Total of 20 Healthy Volunteers Served as Matched Controls who Were Matched for age and BMI to Patients With OSA to Minimize Confounding Factors. Inclusion and Exclusion Criteria Were the Same for all the Study Groups.

### Inclusion Criteria

A. Age 18–65 years old.
B. No gender limitation.

### Exclusion Criteria

A. Age < 18 years old or > 65 years old.
B. History of or active cancer.
C. Severe cardiopulmonary disease.
D. Infectious disease.
E. History of acute upper respiratory tract infection in the past month: nasal congestion, sneezing, salivation, etc.
F. History of any prescribed medications.
G. Did not obtain the consent of the subject.

### Polysomnography

Apnea-hypopnea index (AHI) was calculated as the number of apneas and hypoventilation per hour during sleep time. We also recorded the lowest saturation oxygen (LSaO_2_) as an indicator of nocturnal hypoxemia. Based on the AHI, subjects were divided into control group (AHI < 5, *n* = 20) and OSA group (AHI > 5, *n* = 74). The OSA group was assigned as follows: Mild, AHI: 5-14.9 (*n* = 15), moderate AHI:15.0–29.9 (*n* = 24), and severe, AHI ≥ 30 (*n* = 35).

### Human Serum Samples Collection

Serum samples (approximately 3 mL) were collected from the antecubital inferior caval vein, allowed to clot for 30 min, and centrifuged at 3,000 g for 10 min at 25°C. The resulting serum was aliquoted and frozen at −80°C for further analysis.

All experimental procedures were approved by the Second Xiangya Hospital of Central South University and were in accordance with the laboratory guidelines.

### Clinical Laboratory Measurements

Clinical laboratory parameters including age, sex, alanine aminotransferase (ALT), aspartate aminotransferase (AST), total protein (TP), albumin (ALB), globulin (Glo), and hemoglobin (HGB) were measured and recorded on admission. Biochemical tests were performed using a routine automated analyzer (HIitachi 7180). The concentrations of the four serum fibrosis markers PIIINP, CIV, LN, and HA were determined by electrochemiluminescence immunoassay (Mindray CL-i2000). Serum Chitinase 3-like protein 1 was measured by electrochemiluminescence immunoassay (YHLO iFlash 3000-H).

### Cell Culture

LX-2, human immortalized hepatic stellate cell line (Hunan, China) was cultured in high glucose DMEM supplemented with 10% fetal bovine serum (FBS) and 1% penicillin/streptomycin. The cells were starved for 10 h in DMEM medium containing 1% FBS before treatment.

### LX-2 Intermitted Hypoxia *in vitro*

LX-2 were grown and maintained in growth medium (EGM-2-MV; Clonetics) containing depleted fetal bovine serum and further cultured at 37°C and 5% CO_2_ in a cell culture incubator. Cells were grown in T-25 flasks and incubated in hypoxia (0.1–21% O_2_, equilibrated N_2_) or normoxia (21–21% O_2_) every 5 min for 3–7 days.

### Western Blotting

Intracellular proteins were Extracted employing RIPA buffer and PMSF (Beyotime, Shanghai, China) at 99:1 ratio. And total protein was quantified with a BCA kits (Thermos Fisher Scientific, USA). Then, equal amounts of protein were separated by SDS-PAGE (10% gels), and transferred to PVDF membranes (Sigma-Aldrich, USA). After blocking with 5% skim milk for 1 h, the membranes were incubated with the primary antibodies overnight at 4°C. The next day, the membranes were incubated at room temperature for 1 h with specific secondary antibodies. The membranes were rinsed following by chemiluminescence imaging. Quantitative analysis of Western blots was conducted by ImageJ software. Antibodies: FN (Abcam, #ab45688, 1:1000), COL1A1 (CST, #72026, 1:1000), MMP9 (Abcam, #EP1254, 1:1000), CHI3L1 (Abcam, # ab255297, 1:1000), beta-actin (Sangon, #D110001, 1:2500). Image J was used to quantify the gray values for each target proteins bands.

### Statistical Analysis

Statistical Package for Social Sciences (SPSS) software version 23.0 was used for statistical analysis. Patient characteristics were expressed as mean ± standard deviation, median, and range or proportion. One-way ANOVA was used to compare all continuous variables between groups, and Student's test was performed to assess the significance of differences between the two groups. The Wilcoxon rank sum test was used for non-parametric data. Correlations were assessed using Spearman's correlation coefficient for continuous variables and Kendall's correlation coefficient for categorical variables. For 95% confidence interval, a *p*-value < 0.05 considered to be statistically significant.

## Results

### Demographic, Clinical, and Biochemical Profile

The clinical characteristics and biochemical parameters of the study subjects have been summarized in Table 1. It could be found that the control group and the OSA groups did not have significant differences in anthropometric data including age, male-female ratio, and BMI. Mean values of AHI (*p* = 0.001), LSaO_2_ (*p* = 0.003) were significantly different in all subject groups.

**Table 1:** Demographics characteristics and biochemical profiles of study subjects.

**Variables**	**Control (*n* = 20)**	**OSA (*****n*** **=** **74)**		
		**Mild OSA (*n* = 15)**	**Moderate OSA (*n* = 24)**	**Severe OSA (*n* = 35)**
Age (years)	45.08 ± 13.73	46.12 ± 8.3	45.45 ± 9.51	44.2 ± 11.47
Sex (male/female)	17/3	12/3	20/4	29/6
BMI (kg/m^2^)	28.74 ± 11.43	29.21 ± 8.99	29.45 ± 7.52	30.27 ± 12.53
AHI (events/h)	2.1 (1.5, 3)	11.35 (8.2, 18.6)^*^	24.2 (21.1, 31.5)^*^^#^	59.4 (47.35, 76.1)^*^^#^^&^
LSaO_2_ (%)	94.8 ± 2.9	81.5 ± 4.3^*^	74.5 ± 11.8^*^^#^	55.2 ± 5.9^*^^#^^&^
ALT (U/L)	11 (9.3, 20)	12.1 (4.3, 34.1)	24.6 (19, 37.4)^*^^#^	30.91 (15.1, 45.8)^*^^#^
AST (U/L)	14.9 (13.4, 21.3)	15.4 (10.2, 24.3)	19.5 (16.7, 25.2)^*^^#^	22.6 (10.1, 30.5)^*^^#^
ALT/AST	1.22 (0.71, 1.38)	1.12 (0.61, 1.54)	0.74 (0.58, 1.01)^*^^#^	0.72 (0.51, 1.54)^*^^#^
TP (g/L)	63 (60.45, 67.65)	64.1 (59.2, 68.5)	67.5 (64.8, 71)	66.2 (59.8, 75.2)
ALB (g/L)	38.4 (36.5, 42.4)	39.2 (35.1, 43.2)	41.75 (39.38, 43.7)^*^	40.15 (38.12, 45.3)^*^
Globulin (g/L)	24.9 (22.75, 28.43)	25.3 (21.45, 29.22)	26.3 (23.5, 29.1)	26.2 (24.5, 30.5)
A/G	1.6 ± 0.23	1.59 ± 0.21	1.61 ± 0.27^*^	1.62 ± 0.25^*^
HGB (g/L)	133.3 ± 19.63	134.2 ± 12.21	147.6 ± 14.48^*^^#^	152.9 ± 13.52^*^^#^

*AHI, apnea-hypopnea index; BMI, body mass index; LSaO_2_, lowest saturation oxygen; ALT, alanine aminotransferase; AST, aspartate aminotransferase; TP, total protein; ALB, albumin; HGB, Hemoglobin*.

* *p < 0.05, compared with the control group*.

# *p < 0.05, compared with the mild OSA group*.

& *p < 0.05, compared with the moderate OSA group*.

Comparison of hepatic function index between four groups: The concentrations of serum ALT, AST, ALT/AST, plasma HGB in moderate OSA patients, severe OSA groups was significantly higher than control and mild OSA groups. However, the ALB, A/G concentration in moderate OSA, severe OSA groups was significantly higher than control group.

### Four Serum Liver Fibrosis Markers and CHI3L1 Levels in Patients With OSA and Control Subjects

Serum HA levels were significantly higher in the OSA group compared with the control group (*p* < 0.05) (Table 2). In addition, serum HA levels progressively increased with increasing severity of OSA (33.12 ± 10.6 ng/mL for mild OSA, 34.53 ± 13.2 ng/mL for moderate OSA, and 35.44 ± 10.7 ng/mL for severe OSA) (Table 3).

**Table 2:** Serum levels of 4 serum markers and CHI3L1 in the subjects.

**Variables**	**Control**	**OSA**	***p* value**
	**(*n* = 20)**	**(*n* = 74)**	
HA (ng/ml)	23.62 ± 5.93	34.42 ± 8.47	0.001^*^
PIIINP (ng/ml)	7.384 ± 1.69	7.69 ± 2.75	0.524
CIV (ng/ml)	41.78 ± 8.72	49.65 ± 6.43	0.004^*^
LN (ng/ml)	27.29 ± 6.39	28.63 ± 7.68	0.341
CHI3L1 (ng/ml)	34.45 (26.7, 41.3)	39.64 (31.2, 55.9)	0.03^*^

*HA, hyaluronan; PIIINP, procollagen type III N-terminal peptide; CIV, type IV collagen; LN, laminin; CHI3L1, Chitinase-3-like protein 1*.

* *p < 0.05, OSA group compared with the control group*.

**Table 3:** Four serum markers and CHI3L1 in the control and OSA subgroups.

**Variables**	**Control (*n* = 20)**	**OSA (*****n*** **=** **74)**		
		**Mild OSA (*n* = 15)**	**Moderate OSA (*n* = 24)**	**Severe OSA (*n* = 35)**
HA (ng/ml)	23.62 ± 5.93	33.12 ± 10.6^*^	34.53 ± 13.2^*^	35.44 ± 10.7^**^
PIIINP (ng/ml)	7.384 ± 1.69	7.12 ± 2.28	7.368 ± 2.24	8.32 ± 3.1
CIV (ng/ml)	41.78 ± 8.72	37.41 ± 4.57	39.55 ± 3.46	45.52 ± 2.45^*^
LN (ng/ml)	27.29 ± 6.39	27.37 ± 7.69	29.12 ± 7.92	30.48 ± 7.4
CHI3L1 (ng/ml)	34.45 (26.7, 41.3)	35.42 (29.42, 41.2)	37.88 (29.9, 51.2)	40.35 (31.9, 54.9)^*^

*HA, hyaluronan; PIIINP, procollagen type III N-terminal peptide; CIV, type IV collagen; LN, laminin; CHI3L1, Chitinase-3-like protein 1*.

* *p < 0.05, compared with the control group*.

** *p < 0.001, compared with the control group*.

A similar trend was observed for serum CIV and CHI3L1 levels. serum CIV and CHI3L1 levels were higher in the OSA group (*n* = 74) than in the control group, with a significant difference between the severe OSA and control groups (41.78 ± 8.72 vs. 45.52 ± 2.45 ng/ml, 34.79 ± 12.08 ng/ml vs. 60.9 ± 7.2 ng/ml, *p* < 0.05).

### Correlations Between Serum Markers of Liver Fibrosis and Polysomnographic Parameters

**Figure 1:**
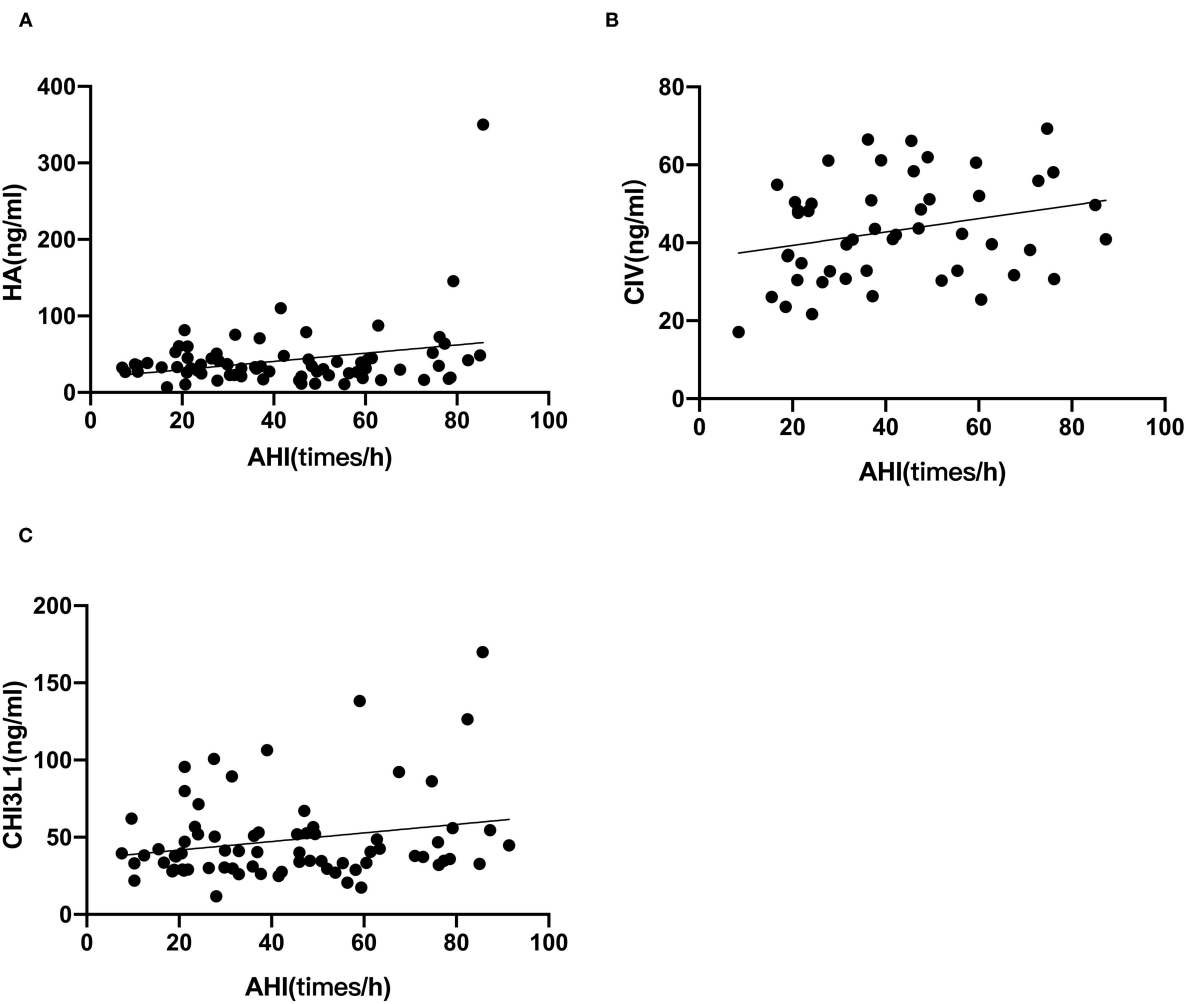
AHI correlated with liver fibrosis serum biomarkers in patients with OSA. The correlations between AHI and each marker **(A)** HA; **(B)** CIV; **(C)** CHI3L1 were assessed by Spearman's rank correlation coefficient. The *p*-values and correlation coefficients are shown in each plot.

**Figure 2:**
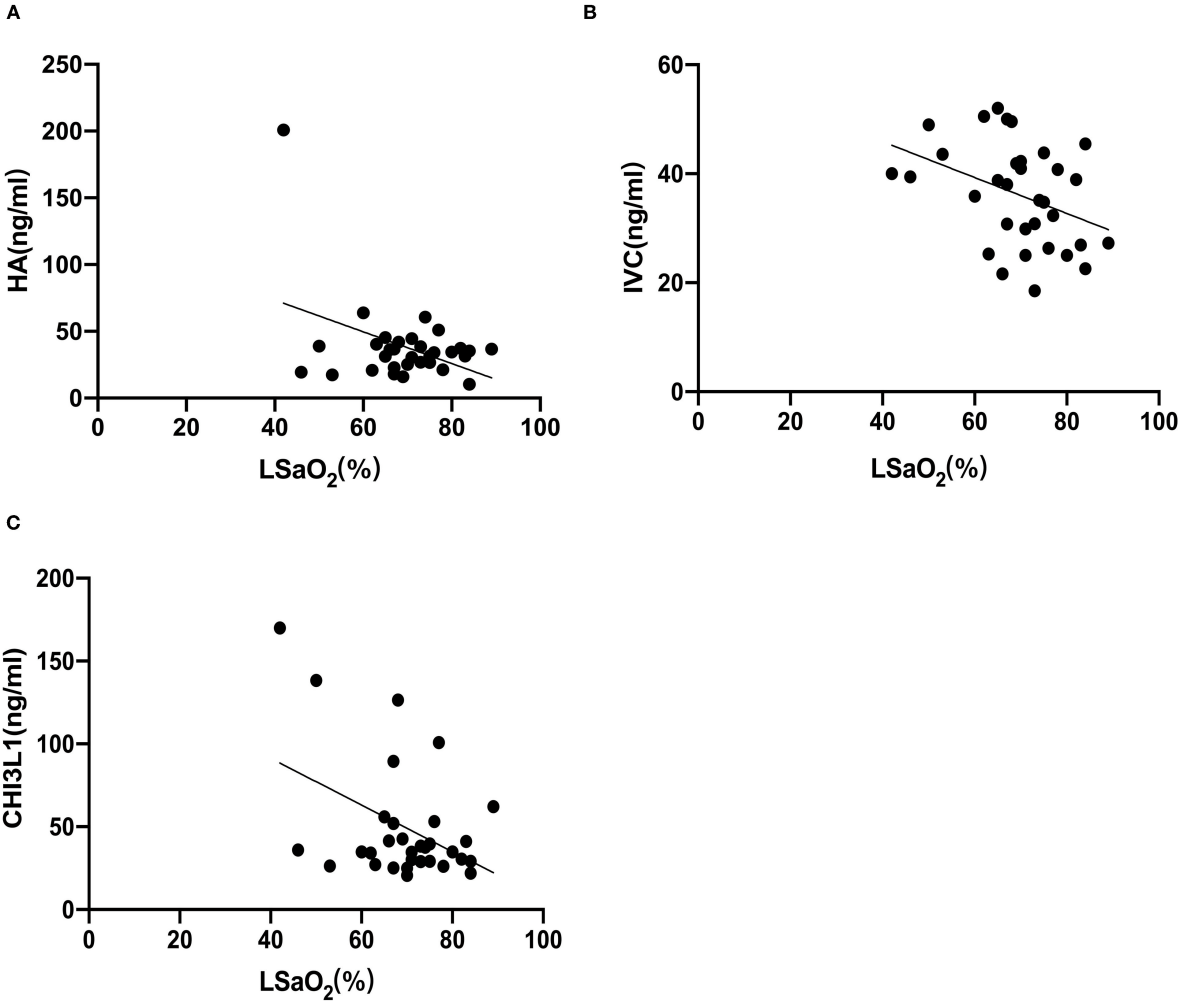
LSaO_2_ correlated with liver fibrosis serum biomarkers in patients with OSA. The correlations between LSaO_2_ and each marker **(A)** HA; **(B)** CIV; **(C)** CHI3L1 were assessed by Spearman's rank correlation coefficient. The *p*-values and correlation coefficients are shown in each plot.

Based on the foregoing results, we found that the HA, CIV and CHI3L1 may be used as an indicator of liver fibrosis in OSA patients. By analyzing the total population of OSA patients (*n* = 74), significant correlations were observed between three liver fibrosis serum markers levels and several polysomnographic parameters including AHI, LSaO_2_, as shown in Table 4, Figures 1, 2. Specifically, serum liver fibrosis markers HA (*r* = 0.2694, *p* = 0.008) and CIV (*r* = 0.2693, *p* = 0.03) levels were positively correlated with AHI, but negatively correlated with LSaO_2_ (*r* = −0.4081, *p* = 0.01; *r* = −0.426, *p* = 0.007) respectively. Similarly, serum CHI3L1 levels showed positive correlation with AHI (*r* = 0.221, *p* = 0.02), and negative correlation with LSaO_2_ (*r* = −0.3814, *p* = 0.01), respectively.

**Table 4:** Correlation analysis between different parameters in OSA patients.

	**AHI**		**LSaO**_**2**_	
	***r***	***P*-value**	***r***	***P-*value**
HA	0.2694	0.008	−0.4081	0.01
CIV	0.2693	0.03	−0.426	0.007
CHI3L1	0.221	0.02	−0.3814	0.01

*AHI, apnea-hypopnea index; LSaO_2_, lowest saturation oxygen; HA, hyaluronan; PIIINP, procollagen type III N-terminal peptide; CIV, type IV collagen; LN, laminin; CHI3L1, Chitinase-3-like protein 1*.

### Effect of Intermittent Hypoxia on Fibrotic Markers

**Figure 3:**
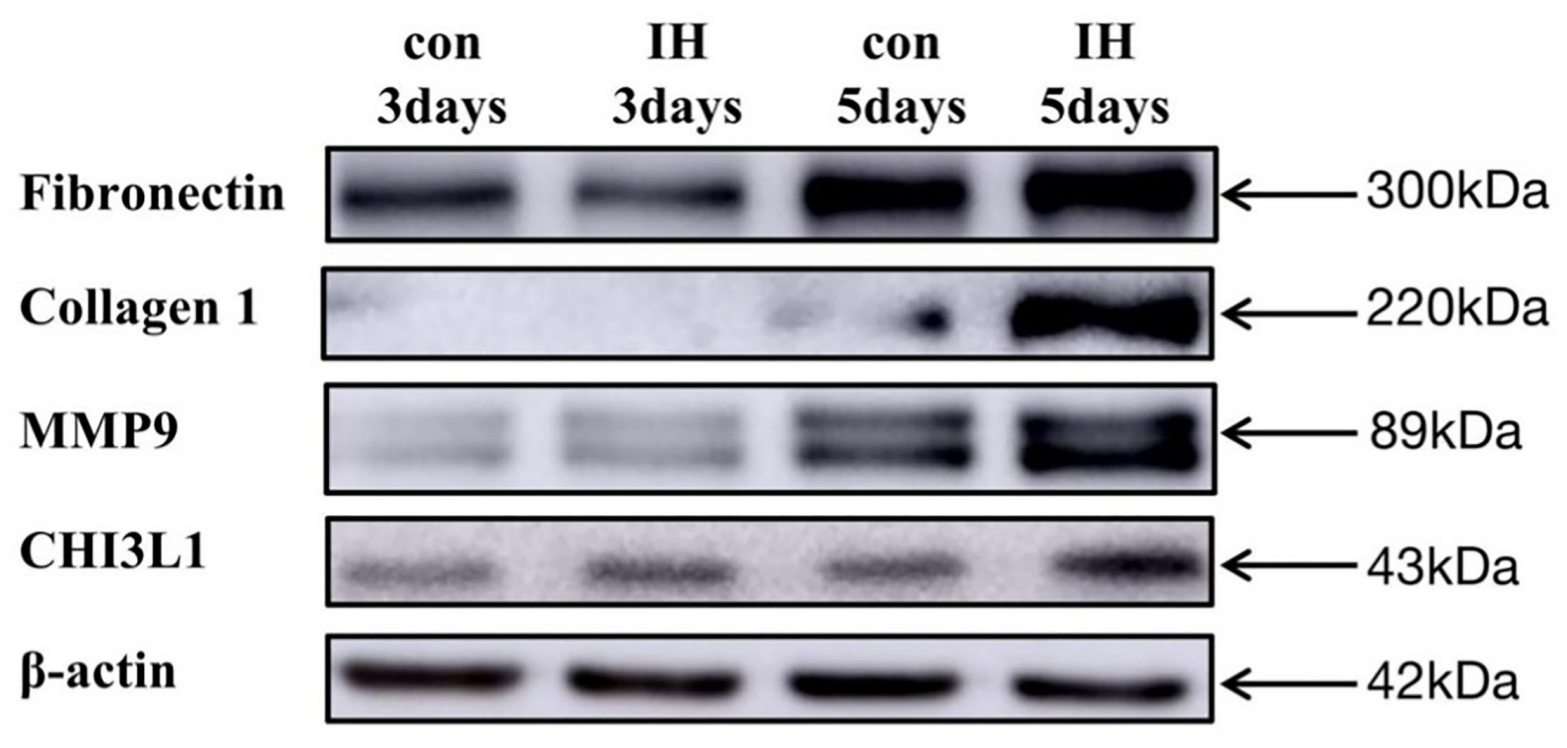
LX-2 cell changes in FN, Collagen 1, MMP9, CHI3L1 expression *in vitro* exposures to IH (1–21% O_2_) or to RA (21–21% O_2_) for 1–5 days.

LX-2 cell exposed to IH for 3, 5 days showed significant FN, Collagen, MMP9 increase in compared to normoxia. Similarly, IH exposures for 5 days resulted in significant increases in CHI3L1 expression (Figure 3).

## Discussion

In the present study, we investigated changes in the levels of five serum liver fibrosis markers in patients with OSA and found that serum HA, CIV and CHI3L1 levels were significantly increased in patients with compared with healthy volunteers. In addition, serum HA, CIV and CHI3L1 levels were strongly correlated with AHI and LSaO_2_ in OSA patients, which suggested that three serum markers levels were related to OSA severity. In addition, intermittent hypoxia *in vitro* can increase expression of fibrotic markers in hepatic stellate cell. Therefore, OSA might either directly or indirectly trigger or exacerbate liver fibrosis, possibly via oxidative stress-related pathways.

As shown Table 2, it is obviously appeared that OSA patients have hepatic impairment. And as the degree of OSA increases, the more severe the liver function impairment. But as liver damage gets worse, the hepatitis can progress to liver fibrosis. There are some experimental and clinical data have suggested that OSA may contribute to the development and exacerbation of liver fibrosis (5, 6). However, the diagnosis of liver fibrosis mainly based on liver biopsy, which is regarded as the gold standard. However, liver biopsy requires coarse needle puncture, which is invasive (20). It is necessary to substitute liver biopsy with non-invasive techniques. Until now, there are two methods used in clinical scenario: one is ultrasound detection the other is serum indicators. However, transient ultrasound elastography (Fibro scan) and shear wave elastography (SWE) are influenced by many factors, such as ascites, obesity, operator experience, etc., Therefore, non-invasive serum fibrosis markers were chosen in this study. Serum HA, LN, PIIINP, and CIV have been used as serological markers to diagnose hepatic fibrosis, estimate the severity of the condition, and assess the prognosis for patients with chronic hepatic disease with better specificity and sensitivity (21).

**Figure 4:**
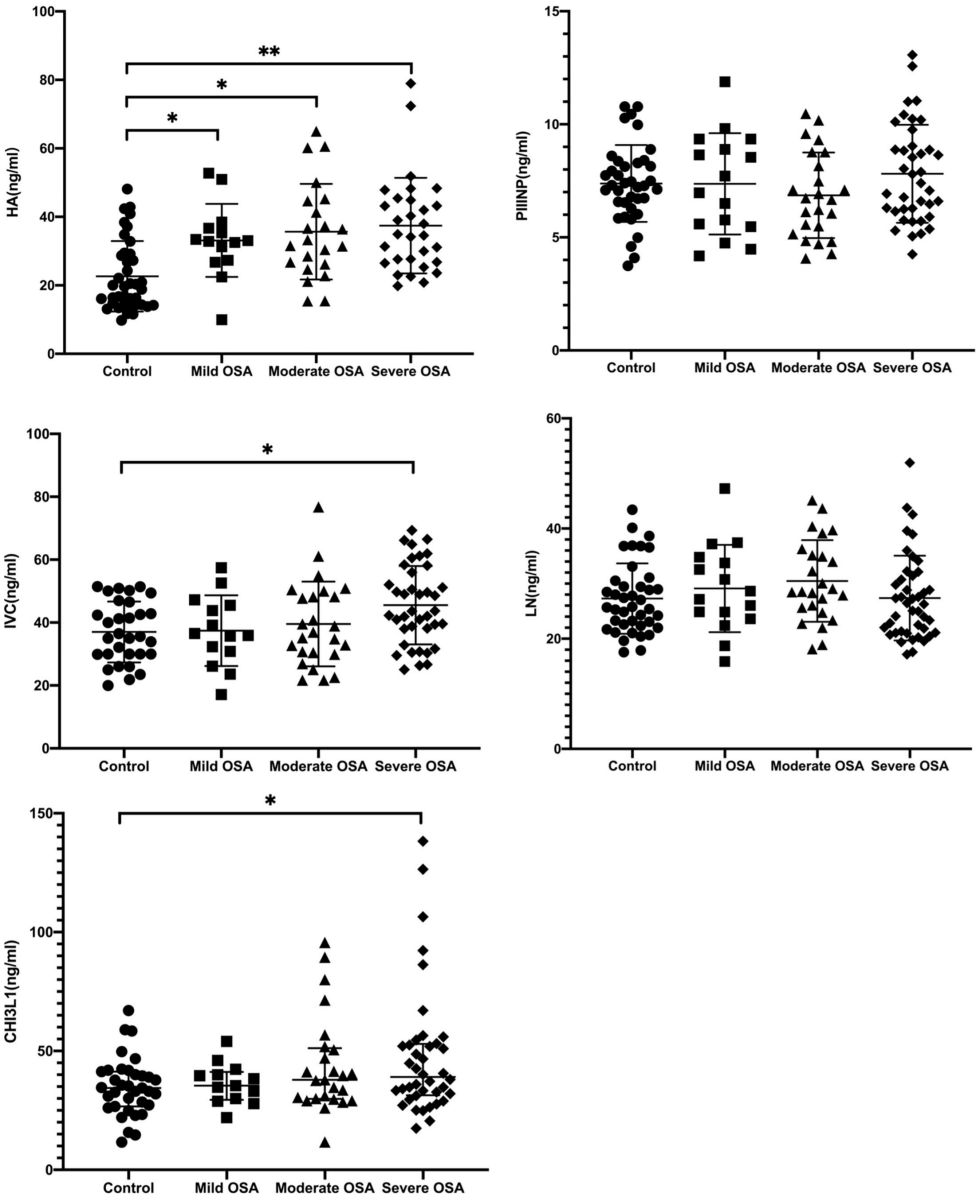
Serum HA, PIIINP, CIV, LN, and CHI3L1 levels in patients with OSA. *p < 0.05, **p < 0.001, by Kruskal-Wallis test with Dunn's multiple comparison test.

From Figure 4 and Table 2 it is clear that HA is the most sensitive index among the five liver fibrosis serum markers. Cai et al. suggested that HA has a strong positive correlation with the degree of hepatic fibrosis compared with LN, PIIINP, and CIV (22). This is consistent with the results of our previous study on the relationship between serum fibrosis markers and liver histological changes (23). The concentration of CIV might increase during the early stages of hepatic fibrosis and could promote capillarization of hepatic sinuses. Specifically, serum liver fibrosis markers HA, CIV levels were positively correlated with AHI, but negatively correlated with LSaO_2_, respectively. Therefore, HA, CIV can reflect to some extent that OSA patients may tend to have liver fibrosis, and it will deepen with the degree of OSA. To our knowledge, this is the first study on changes in serum liver fibrosis marker levels in patients with OSA, and these results suggest that HA, CIV may serve as a potential surrogate biomarker for the severity of various chronic hypoxic liver diseases.

In present study, we found that PIIINP and LN these two serological markers were not significantly different in healthy subjects vs. OSA patients (Table 3). Compared with other two serological indexes, these two indexes have poor sensitivity and specificity in diagnosing liver damage. It is reported that PIIINP has reached a limited clinical application with 76–78% sensitivity and 71–81% specificity (24). Moreover, LN could only be a predictor of portal hypertension (25), which is not a liver specific biomarker and has not gained widespread acceptance.

CHI3L1 is a novel serological marker for the assessment of liver fibrosis compared to the other four traditional serological markers. It is involved in physiological and pathophysiological processes and has been shown to be a common inflammatory cytokine that contributes to OSA (11). It is also reported that plasm CHI3L1 levels are associated with hypertension in patients with OSA (26). However, CHI3L1 is not only important in inflammation and cardiovascular disease but also plays a role in liver fibrosis. Some studies demonstrated that CHI3L1 is liver-enriched and has better application value in staging liver fibrosis than platelet ratio index (APRI) and fibrosis-4 index (FIB-4). Therefore, our study used CHI3L1 to evaluate the extent of liver fibrosis in OSA patients.

In addition, there was a positive correlation between CHI3L1 levels and AHI and a negative correlation between CHI3L1 levels and LSaO_2_. These results suggest that serum CHI3L1 levels may be a modifying factor in the development of OSA and may serve as a potential biomarker of OSA severity.

The above results suggest that clinicians should monitor serum HA, CIV, CHI3L1 levels in OSA patients and judge the liver damage by combining the levels of AHI and LaSO_2_, to select the appropriate treatment plan as soon as possible.

Since previous studies have shown that OSA and its associated intermittent hypoxia (IH) induce oxidative stress. Therefore, we speculated that OSA may be inducing liver fibrosis through IH. Several studies have shown that intermittent hypoxemia caused by OSA can accelerate the progression of liver fibrosis. For example, chronic intermittent hypoxia accelerates hepatic fibrosis in rats with combined hypoxia and non-alcoholic steatohepatitis through angiogenesis (27). Another study also revealed that TLR4 mediates inflammation and hepatic fibrosis induced by chronic intermittent hypoxia in rats (28). Previous studies have investigated the effects of IH on liver fibrosis in animal models, but hepatic stellate cells also play critical roles in the development of liver fibrosis. To the best of our knowledge, this is the first study on LX-2 exposed to IH. Another important result of our study is LX-2 exposed to IH showed significant increases in FN, MMP9, COL1A1 expression. All three of the above proteins are conventional proteins associated with liver fibrosis. In contrast, CHI3L1 is a relatively novel protein, and its elevated expression in IH exposure is consistent with the previous clinical findings. Thus, it is highly likely that OSA, a systemic disease which activates a multiplicity of pathophysiological pathways, will exert its detrimental endo-organ effects via a large number of mechanisms.

The above results suggest that CHI3L1 may be useful as a biomarker of liver fibrosis in patients with OSA. Although CHI3L1 appears promising as a serum biomarker in this limited trial and may also provide a mechanistic link to the pathogenesis of liver fibrosis, further testing is needed to validate its use in the routine management of patients with liver fibrosis.

Our findings have several limitations. The first and most significant limitation of our study revolves around the small sample size. Obviously, in this context we advocate caution in interpreting our data and studying these findings further in a larger cohort. Second, the evaluation of liver fibrosis severity in our experiment only has serum testing, it is also necessary to prepare a combined ultrasound as well as CT for imaging aspects of the test and to rigorously design liver puncture examinations in patients with OSA. Finally, our study only analysis the role of intermittent hypoxia *in vitro*, without performing any *in vivo* experiments. But the strength of the vitro experiments in our study could be considered as a starting observation for *in vivo* studies. Therefore, further basic mechanistic studies are needed.

## Conclusion

In conclusion, our findings suggest that serum HA, CIV, CHI3L1 levels are related to the severity of OSA in patients. Moreover, hepatic stellate cell LX-2 *in vitro* data demonstrate that IH causes fibrotic protein over-expression. These findings indicate that OSA may induce liver fibrosis, which may be through IH. Although the detailed mechanisms and clinical implications remain to be elucidated in the future.

## Data Availability Statement

The original contributions presented in the study are included in the article/Supplementary Material, further inquiries can be directed to the corresponding author/s.

## Ethics Statement

The studies involving human participants were reviewed and approved by Second Xiangya Hospital of Central South University (No. 2021168). The patients/participants provided their written informed consent to participate in this study.

## Author Contributions

JC: formal analysis, investigation, and writing—original draft. XL: methodology and data curation. PH: data curation and software. SL and FW: investigation. RC: data curation. ZC and LZ: visualization. MS: software. MH: supervision and writing—review and editing. All authors contributed to the article and approved the submitted version.

## Conflict of Interest

The authors declare that the research was conducted in the absence of any commercial or financial relationships that could be construed as a potential conflict of interest.

## Publisher's Note

All claims expressed in this article are solely those of the authors and do not necessarily represent those of their affiliated organizations, or those of the publisher, the editors and the reviewers. Any product that may be evaluated in this article, or claim that may be made by its manufacturer, is not guaranteed or endorsed by the publisher.
