# Supplementary figures and images for: Increased Levels of CHI3L1 and HA Are Associated With Higher Occurrence of Liver Damage in Patients With Obstructive Sleep Apnea
Source: Front Med (Lausanne). 2022 Feb 25;9:854570. doi: 10.3389/fmed.2022.854570 (PMC8913888; doi:10.3389/fmed.2022.854570)

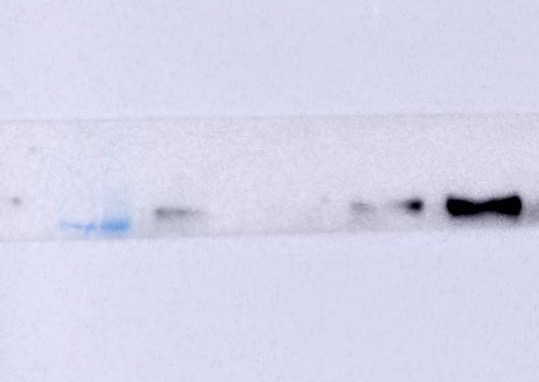

Supplement: Supplementary file 1 [file Data_Sheet_1.ZIP › original gels/col 70s 2021.05.22_13.32.31_Ch+Marker.jpeg]

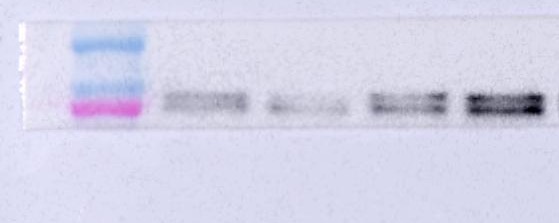

Supplement: Supplementary file 1 [file Data_Sheet_1.ZIP › original gels/mmp9 2021.05.22_13.15.10_Ch+Marker.jpeg]

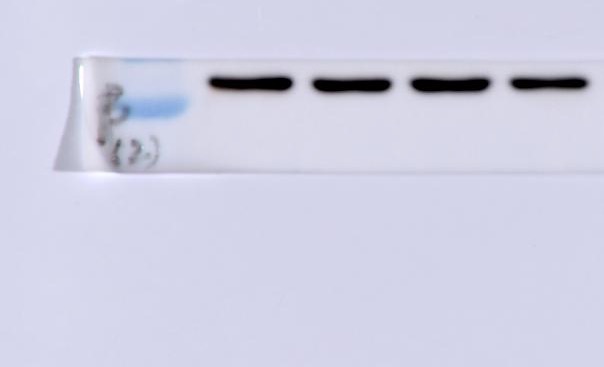

Supplement: Supplementary file 1 [file Data_Sheet_1.ZIP › original gels/actin 3s 2021.05.22_14.02.39_Ch+Marker.jpeg]

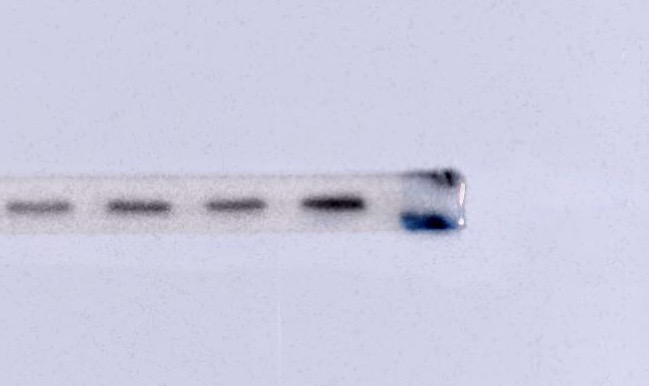

Supplement: Supplementary file 1 [file Data_Sheet_1.ZIP › original gels/CHI3L1 2s 2021.05.22_19.06.14_Ch+Marker.jpeg]

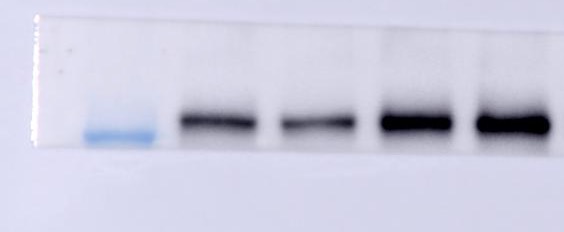

Supplement: Supplementary file 1 [file Data_Sheet_1.ZIP › original gels/fn 3s 2021.05.22_13.08.57_Ch+Marker.jpeg]
